# Convergent evolution of floral signals underlies the success of Neotropical orchids

**DOI:** 10.1098/rspb.2013.0960

**Published:** 2013-08-22

**Authors:** Alexander S. T. Papadopulos, Martyn P. Powell, Franco Pupulin, Jorge Warner, Julie A. Hawkins, Nicolas Salamin, Lars Chittka, Norris H. Williams, W. Mark Whitten, Deniz Loader, Luis M. Valente, Mark W. Chase, Vincent Savolainen

**Affiliations:** 1 Imperial College London, Silwood Park Campus, Ascot, Berkshire SL5 7PY, UK; 2 Jodrell Laboratory, Royal Botanic Gardens, Kew, Richmond, Surrey TW9 3DS, UK; 3 Centre for Plant Diversity and Systematics, School of Plant Sciences, University of Reading, Reading, Berkshire RG6 6AS, UK; 4 Lankester Botanical Garden, University of Costa Rica, PO Box 1031-7050, Cartago, Costa Rica; 5 Department of Ecology and Evolution, Biophore, University of Lausanne, Dorigny, Lausanne 1015, Switzerland; 6 Biological and Experimental Psychology, School of Biological and Chemical Sciences, Queen Mary University of London, Mile End Road, London E1 4NS, UK; 7 Florida Museum of Natural History, University of Florida, Gainesville, FL 32611-2710, USA

**Keywords:** convergent evolution, Oncidiinae, deceptive pollination, Neotropical plant communities, insect colour vision

## Abstract

The great majority of plant species in the tropics require animals to achieve pollination, but the exact role of floral signals in attraction of animal pollinators is often debated. Many plants provide a floral reward to attract a guild of pollinators, and it has been proposed that floral signals of non-rewarding species may converge on those of rewarding species to exploit the relationship of the latter with their pollinators. In the orchid family (Orchidaceae), pollination is almost universally animal-mediated, but a third of species provide no floral reward, which suggests that deceptive pollination mechanisms are prevalent. Here, we examine floral colour and shape convergence in Neotropical plant communities, focusing on certain food-deceptive Oncidiinae orchids (e.g. *Trichocentrum ascendens* and *Oncidium nebulosum*) and rewarding species of Malpighiaceae. We show that the species from these two distantly related families are often more similar in floral colour and shape than expected by chance and propose that a system of multifarious floral mimicry—a form of Batesian mimicry that involves multiple models and is more complex than a simple one model–one mimic system—operates in these orchids. The same mimetic pollination system has evolved at least 14 times within the species-rich Oncidiinae throughout the Neotropics. These results help explain the extraordinary diversification of Neotropical orchids and highlight the complexity of plant–animal interactions.

## 1. Introduction

Competition for pollinators in tropical plant communities is considerable as many angiosperms require animal vectors for pollination [1,2]. A variety of floral signals are used to entice pollinating animals, with shape [3], colour [4–7] and scent [8] all playing a role. Although generalist pollination systems are frequent on a global scale [9], specialization of pollination systems is common in the tropics [10] and may have been integral to angiosperm diversification [9]. Plants have achieved this specialization through modification of floral signals and even the use of unusual rewards, such as oils or resins [9,11]. Adaptation to a guild of pollinators that share a functional role (e.g. pollination by birds [12]) is more widespread than specialization to a single species [9]. Dependence on functionally similar pollinators has driven convergent evolution of floral signals, whereby similar floral traits have arisen in distantly related taxa [12], although some studies have questioned the ‘pollination syndrome’ concept [13,14].

Pollination of specialized plants is often pollinator limited in tropical communities [15,16]. This is pronounced in members of the orchid family (Orchidaceae), which possess highly specialized pollination mechanisms [17,18]. Orchidaceae is one of the largest families of angiosperms, comprising as many as 25 000 species [17]. Approximately 8000–10 000 of these species offer no floral reward, yet they rely on animal pollination [18,19]. Some orchids use food-deception to attract pollinators by imitating floral signals of rewarding plants either directly (via Batesian mimicry [20] or convergence on the floral signals of rewarding species) or indirectly (non-model mimicry [17]). Batesian floral mimicry and convergence occur when selection drives a non-rewarding mimic species to resemble a rewarding floral model to attract the signal receiver [6,21–23]. Non-model mimicry systems [17] do not require a specific model species to be imitated, but instead more general floral features of co-occurring plants are displayed by the non-rewarding plant species. In this case, non-rewarding orchids exploit the food-foraging behaviour or perceptual biases of naive pollinators and, because they are not reliant on specific model species, are more dependent upon the species richness and abundance of the rewarding community [19,24,25].

**Figure 1.**
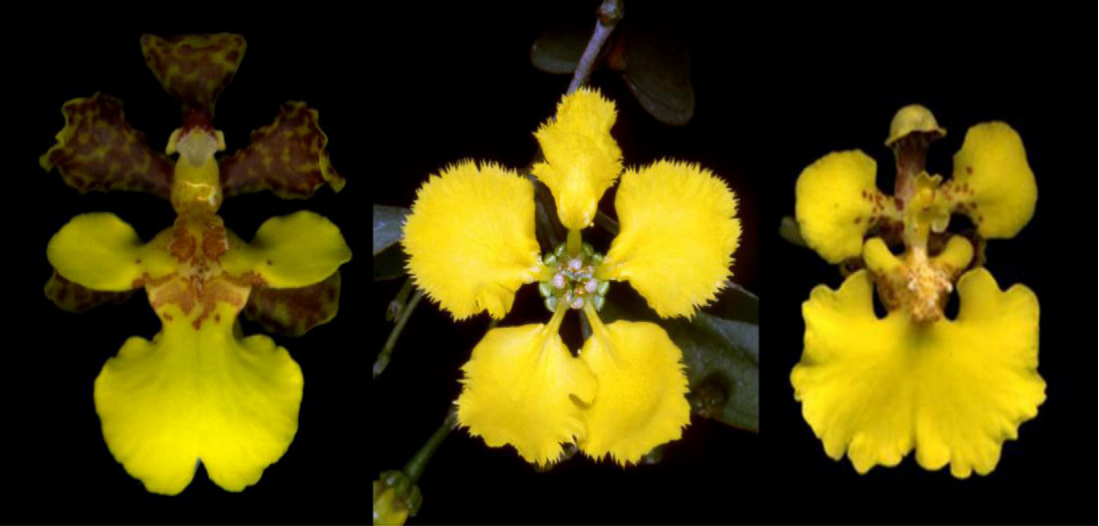
Floral resemblance of *Stigmaphyllon* sp. (centre; Malpighiaceae) and Oncidiinae orchids *Trichocentrum ascendens* and *Rossioglossum ampliatum* (left and right; Oncidiinae: Orchidaceae).

We focus our study on the highly diverse orchid subtribe Oncidiinae (tribe Cymbidieae). Widespread in tropical America, they comprise over 1700 species in 60 genera [26–28]. The majority of these species are non-rewarding and self-incompatible, presumably attracting pollinators through some form of deception. Some members of the *Rodriguezia* clade offer nectar rewards [26] and ≈70 species of *Oncidium* produce oils that bees harvest for food or nest construction [29]. There are reports of oil production in other genera [30], but this may not be in sufficient quantity to qualify as a reward [27,31]. ‘Classic’ *Oncidium*-type flowers possess superficially similar floral colour and shape to rewarding Malpighiaceae species (figure 1; [27,28,31–33]). However, evidence for mimicry is anecdotal and floral colours have only been assessed from the perception of humans, rather than of hymenopteran pollinators. Floral scent is considered to be of limited importance for food-deceptive species [34,35].

First, to determine the degree of convergence in floral colour signals, we investigated the floral colour of self-incompatible, rewardless Oncidiinae that appear to mimic the floral signals of rewarding Malpighiaceae [27,28,31]. The putative Malpighiaceae model species used were *Byrsonima crassifolia*, a Neotropical tree ranging from southern Mexico to Paraguay [36–38], and *Stigmaphyllon lindenianum*, a liana distributed from southern Mexico to northern Argentina [38,39]. Both species have yellow, typically malpighiaceous flowers [36,40] and produce abundant quantities of oil through epithelial elaiophores on sepalar glands [29]. Second, we examined the evolutionary history of floral colour, based on an appropriate model of insect colour vision [41] and a new molecular phylogenetic tree for Neotropical orchids. Finally, we assessed the convergence of floral shape within Oncidiinae, using eigenshape morphometric analyses [42].

## 2. Material and methods

### (a) Floral reflectance

To determine the similarity in floral colour of Malpighiaceae and Oncidiinae species, we collected floral reflectance data for 27 orchid species, four Malpighiaceae and 210 other angiosperm species across 23 sites in Costa Rica (see the electronic supplementary material, table S1). We also collected floral reflectance data from fresh material for a further 63 Oncidiinae and three Malpighiaceae from various sources (see the electronic supplementary material, table S6). Floral reflectance was measured using a miniature Ocean Optics spectrophotometer (Ocean Optics, Ostfildern, Germany), Spectrawin v. 5.0 basic software (Avantes Inc., Broomfield, CO, USA), alongside a D_2_H light source (World Precision Instruments Ltd, Stevenage, UK), which provided UV (300 nm) to red (700 nm) wavelength light—the entire sensitive range of insect spectral perception [43]. Three flowers were collected from all plant species flowering at an accessible height (less than 3 m), within each 500 m^2^ study site. Three floral reflectance measurements were taken from each differently coloured part of each flower. Measurements were taken from the labellum (a modified petal) of all orchid species.

To ascertain how floral colours are perceived by pollinators, particularly hymenopterans, reflectance profiles were converted into colour loci within a colour space using a model of bee colour vision that is applicable to a large number of hymenopteran species [41]. This includes tropical stingless bees, for which UV, blue and green receptors have similar spectral sensitivities across a wide taxonomic range [41,43–45]. Within this colour space, distances between points are indicative of an insect's ability to discriminate between colours. Under natural field conditions, bees of multiple species reliably distinguish differences in colour of more than 0.1 hexagon units, the value below which pollinator constancy does not differ from chance [46,47]. Several alternative models of insect colour vision are available [48,49], the advantages and disadvantages of which have been reviewed elsewhere [49,50]. We used the colour hexagon model [41], because it accurately predicts colour discrimination by multiple different bee species [5,41,51,52].

Reflectance data from individual sites were pooled into three habitat types (see the electronic supplementary material, table S2), and two analyses were performed for each habitat. First, we evaluated whether each orchid species occupied a distinct portion of colour space, that is, whether non-model deception can be ruled out. The median Euclidean distance between the orchid and the rest of the community was compared with the median of all other pair-wise distances, and 1000 bootstrap replicates used to measure significance. Second, we tested whether potential mimics and Malpighiaceae, as a group, occupied a distinct portion of colour space from other species. We assessed whether the mean distance between species occurring within the UV–green portion of colour space was smaller than the mean distance between species in other portions of the colour hexagon; significance was assessed using *t*-tests.

### (b) Phylogenetic inference

A matrix of nuclear internal transcribed spacer DNA sequences for representatives of 211 species of Oncidiinae (plus three outgroup taxa) was assembled from GenBank data and other sequences provided by Norris H. Williams (see the electronic supplementary material, table S3). We used Bayesian phylogenetic reconstruction in MrBayes v. 3.1.2, and the GTR + I + G model of evolution was applied as determined using MrModeltest v. 2.2. The Markov chain Monte Carlo chain was run for 20 million generations, with a 2 million generation burn-in. The floral colour (yellow/non-yellow) of each species was mapped onto the phylogenetic tree using linear parsimony in Mesquite v. 2.73.

### (c) Floral morphology

To determine the role of floral shape in this pollination system, two datasets (described below) were analysed using eigenshape morphometrics [42]. Open curve outlines of each individual's floral shape were captured from digitized images using Media Cybernetics’ Image-Pro Plus v. 6.2 (2006) software and each dataset was analysed separately using Standard Eigenshape v. 2.6 (http://www.morpho-tools.net/). Briefly, coordinates of semi-landmarks interpolated along sample outlines were converted into *φ* shape functions. Singular value decomposition was performed on the covariance matrix of *φ* functions to define eigenshape axes describing shape variation in the sample [42]. This method removes the effect of size, permits analysis of open curves anchored by landmarks and is appropriate for structures to which multiple homologous landmarks are difficult to assign, as for floral shapes [42,53].

The first dataset was used to determine whether the labellum shape of Oncidiinae species was more similar to that of Malpighiaceae than the corolla shape of other angiosperm species found within the 23 study sites. For plants identified to genus or family level in the field, candidate species were determined from Neotropikey (http://www.kew.org/science/tropamerica/neotropikey.htm) and Gargiullo *et al*. [38], matching region, habitat type and elevation of the study site concerned. Images were sourced for 167 species from 46 families (see the electronic supplementary material, table S4). Following eigenshape analysis, Euclidean distances to the nearest Malpighiaceae species, derived from axes describing 90 per cent of the shape variation, were calculated for each species. Significance of differences between two groups (‘yellow-flowered Oncidiinae’ and ‘all other angiosperms’) were assessed using *t*-tests.

To examine floral convergence across the whole Oncidiinae subtribe, 111 images for yellow-flowered species and 158 for non-yellow-flowered species were included in a second dataset (see the electronic supplementary material, table S5). This was composed of photographs of 97 specimens from Lankester Botanical Garden database (www.epidendra.org) and 172 detailed illustrations from ‘The pictorial encyclopedia of *Oncidium*’ [54]. Following eigenshape analysis, we applied canonical variates analysis (CVA) to axes describing 90 per cent of the shape variation, using the *XLSTAT* package for Microsoft-Excel, to determine whether yellow-flowered Oncidiinae could be discriminated from other Oncidiinae by flower shape alone (i.e. to what extent are yellow flowers convergent in shape). In most cases, one flower was analysed per species due to the difficulty in obtaining suitable images, but two flowers were analysed for 32 species with multiple images available (see the electronic supplementary material, table S5). Limited availability of images prevented comprehensive assessment of intraspecific shape variation.

### (d) Additional field observations

A series of field observations were performed to record other aspects of potential mimicry of *B. crassifolia* (Malpighiaceae) by *Trichocentrum ascendens* (Oncidiinae). Bees visiting *B. crassifolia* were caught and examined for the presence of orchid pollinia. Pollinator visitation was also recorded in natural populations of *T. ascendens*. Numbers of individuals, flowers and reproductive success of *T. ascendens* were surveyed in one population with and one without the model *B. crassifolia* (see the electronic supplementary material experimental procedures for locations, study design and observation periods).

## 3. Results

### (a) Floral colour

**Figure 2.**
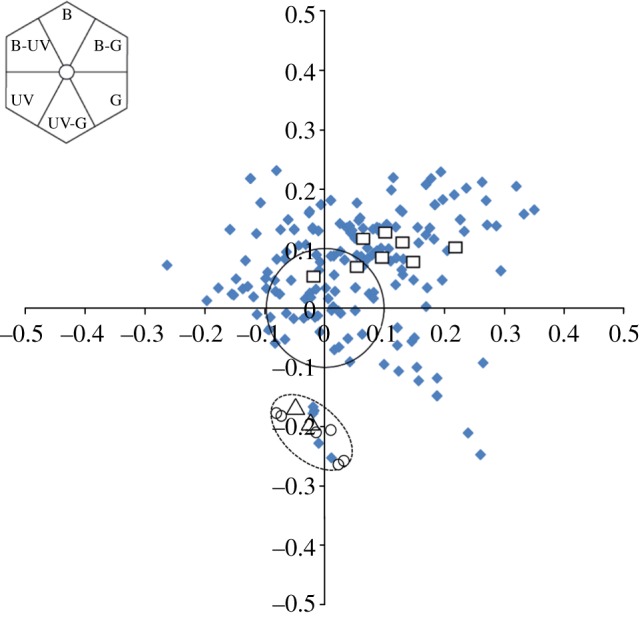
Bee-UV-green Oncidiinae (open circles) and Malpighiaceae species (open triangles) occupy a different portion of bee colour space when compared with the majority of other local flowering species (blue diamonds). Non-bee-UV-green orchids are represented by open squares. Colour loci are calculated according to the hexagon colour model of hymenopteran vision [41]. The inset shows the colour hexagon divided into sections that represent colour names as termed with respect to insect vision (B, blue; G, green; UV, ultraviolet), indicating relative contributions from individual colour receptor types of hymenoptera.

We assessed similarity in floral colour of plants for three habitats from the perspective of insect pollinators using models of colour vision applicable to trichromatic hymenopteran pollinators [41,43]. Within this colour space, yellow-flowered orchids and the two yellow-flowered Malpighiaceae species (*B. crassifolia* and *S. lindenianum*) are all bee-UV-green, that is, they combine long-wavelength reflectance, perceived as yellow by human observers, with UV reflectance [41,55]. The average difference between these yellow orchids and their potential models was 0.04 units, less than the difference detectable by hymenopteran pollinators [41]. This suggests that their floral colours would be perceived as highly similar by these pollinators. Bee-UV-green Oncidiinae or Malpighiaceae were present in 14 sites. In three sites (17, 19 and 21), these analyses showed that two yellow-flowered Oncidiinae species (*T. ascendens* and *Oncidium nebulosum*) match the bee-UV-green colour signal of Malpighiaceae species and occupy a significantly distinct area of colour space from the other species in their communities (figure 2; *p* < 0.001; electronic supplementary material, table S2). Furthermore, orchids within these communities that did not possess bee-UV-green labella were not distinct from local flowering species within the colour hexagon (figure 2). In site seven, sample size was insufficient for a comparison to be made, and in two instances (sites 22 and 23) a second bee-UV-green orchid species was present. In the remaining sites, other bee-UV-green angiosperm species were present.

**Figure 3.**
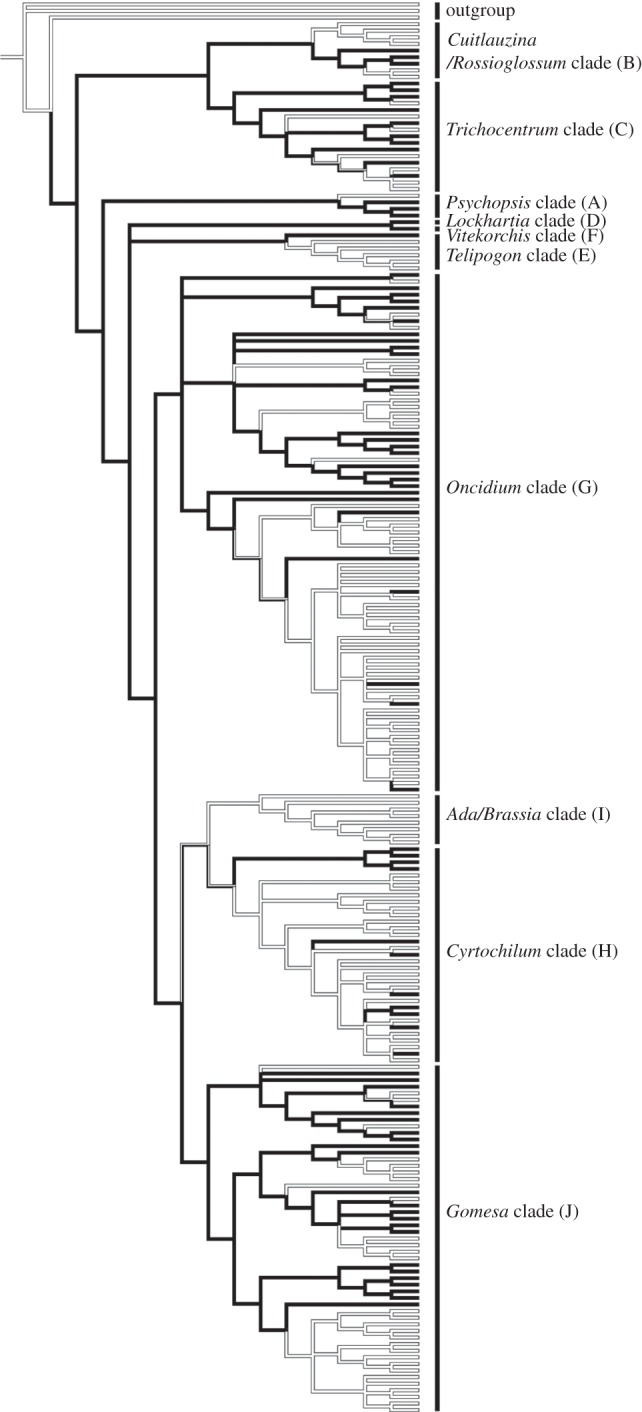
Convergence on a shared area in bee colour space has evolved and been lost multiple times within Oncidiinae. Reproductive strategy, bee-UV-green coloured flowers (black branches) or non-bee-UV-green flowers (white branches), mapped onto the phylogenetic tree of the Oncidiinae. The majority of nodes are well supported (see the electronic supplementary material for details) and agree well with results of Neubig *et al*. [28], who included more taxa and a larger number of DNA regions. Letters in brackets beside the clade name represent the 10 major clades in Oncidiinae [27].

When bee-UV-green orchids and Malpighiaceae species were combined, their floral colour was significantly distinct from the rest of the community in two habitat types (moist forest habitat: *t* = −26.03, d.f. = 91.625, *p* < 0.0001; disturbed habitats: *t* = −9.0534, d.f. = 35.57, *p* < 0.0001). Floral reflectance profiles of the additional orchid samples (i.e. those not observed in the field sites; electronic supplementary material, table S6) confirmed that 70 per cent of the additional orchids and all three Malpighiaceae species are bee-UV-green for insect colour vision and appear yellow to humans. Building on this correlation, we determined (from field observations and the scientific literature) that at least 500 of the 1700 Oncidiinae orchids possess bee-UV-green flowers. Mapping this trait onto a molecular phylogenetic tree of 211 members of the subtribe (see the electronic supplementary material, table S3) demonstrated that this pollination syndrome has evolved independently in at least 14 genera: *Cyrtochilum*, *Erycina*, *Gomesa*, *Lockhartia*, *Miltonia*, *Oncidium*, *Pachyphyllum*, *Psychopsis*, *Otoglossum*, *Rhynchostele*, *Rossioglossum*, *Trichocentrum*, *Tolumnia* and *Zelenkoa* (figure 3; electronic supplementary material, figure S1).

### (b) Floral morphology

**Figure 4.**
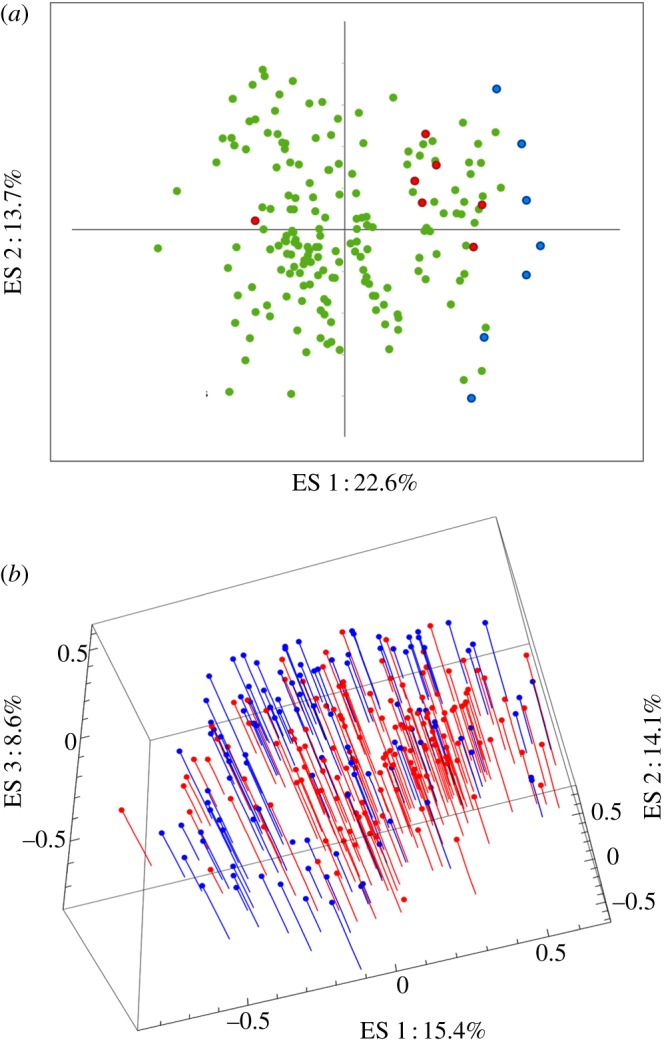
(*a*) Eigenshape (ES) morphometric analysis of Costa Rican angiosperms. Each point represents the position of an individual species in shape space (Malpighiaceae in blue, yellow-flowered Oncidiinae in red and all other angiosperms in green); (*b*) Eigenshape analysis of Oncidiinae labellum shape. Each blue pin represents a yellow-flowered species and each red pin a non-yellow-flowered species.

We examined whether Oncidiinae displaying the yellow colour within these sites had converged on similar floral shapes to those of Malpighiaceae species and whether this was significantly different from the general shape of the rewarding community. Along the first two axes (36% of total shape variation), the shape of Oncidiinae labella is generally more similar to the floral shapes of Malpighiaceae species than to the majority of sympatrically flowering angiosperms, with the exception of *O. nebulosum*, a clear outlier (leftmost red point in figure 4*a*) with respect to other yellow-flowered Oncidiinae. Based on the first 19 eigenshape axes (90% of shape variation), we tested whether the floral shape of yellow Oncidiinae is more similar to Malpighiaceae than other members of the community. The mean distance of yellow-flowered Oncidiinae to the nearest Malpighiaceae species is less than the mean distance of all other angiosperms to the nearest Malpighiaceae species, but the difference is marginally non-significant (*t* = 1.563, d.f. = 6.647, *p* = 0.082). However, when the outlier, *O. nebulosum*, is excluded this difference is highly significant (*t* = 3.583, d.f. = 6.354, *p* = 0.005).

The second morphometric analyses assessed the similarity of 269 Oncidiinae to determine whether floral shape convergence matched that of colour. Although the morphologies of yellow and non-yellow-flowered orchids overlap along the first three axes (38% of the total variation), there is a tendency for unrelated yellow-flowered species to occupy similar areas of morphospace—the same is true for non-yellow-flowered species (figure 4*b*). Discriminant analyses (CVA) of the first 31 axes (90% of shape variation) indicate that the morphological information captured by the labellum outline is sufficient to distinguish ‘yellow’ from non-yellow-flowered species in 78 per cent of cases.

### (c) Shared pollinators and reproductive success

Field observations of hymenopteran visitors to both *B. crassifolia* and *T. ascendens* were of limited success. Several bees were captured on *B. crassifolia*, primarily of the genera *Centris*, *Trigona* and *Paratetrapedia*, but none was carrying orchid pollinia. No pollinators visited *T. ascendens* during the observation periods. As such, confirmation of a shared pollinator was not possible through our observations; however, it has been documented by other studies [29,37,40,56,57]. Our comparative study of two populations of *T. ascendens* demonstrated that female reproductive success in this species was roughly doubled in the presence of *B. crassifolia* (20.4% compared with 8.2%). In the sympatric population, 113 *T. ascendens* flowers were recorded from 31 plants, compared with approximately 320 000 *B. crassifolia* flowers from a single plant. The second population had an equally low number of *T. ascendens* flowers (98 on 23 plants).

## 4. Discussion

### (a) Convergent evolution of floral colour

We show that certain Oncidiinae orchids that conform to the ‘classic’ *Oncidium*-flower type, and *B. crassifolia* and *S. lindenianum* (Malpighiaceae), produce unique floral signals that are so similar that they are unlikely to be distinguished by their hymenopteran pollinators. For two Oncidiinae species, *T. ascendens* and *O. nebulosum*, this shared signal is significantly distinct from the floral reflectance profiles of other neighbouring plant species in bee colour space and is greater than expected by chance. This implies that a visiting bee cannot differentiate between flowers of the two groups with respect to flower colour, which is consistent with both convergent evolution of floral colour to attract similar pollinators and mimicry of oil-bearing Malpighiaceae flowers [27,31–33].

Analysis of other bee-UV-green species within the subtribe revealed that a substantial proportion of species examined possess yellow/bee-UV-green flowers, and that this trait has evolved multiple times, at least 14, within the Oncidiinae (figure 3). Ancestral state reconstruction also indicates that the bee-UV-green flower colour may have been the ancestral condition for the subtribe. Under this scenario, the resemblance to Malpighiaceae has been repeatedly lost and then secondarily regained in numerous lineages. These data provide substantial evidence that not only is convergence on Malpighiaceae flower colour a common feature in Neotropical orchids, but also that it is apparently an evolutionarily labile trait. It seems clear that these species gain a substantial benefit from this convergent coloration; however, it is less clear whether the benefit comes from maintaining reproductive success while conserving resources or as a means to improve fitness through higher outcrossing rates [58].

### (b) Convergence of floral shape

Morphometric analyses demonstrate that convergence in floral shape is also present in Oncidiinae. The extent to which pollinators perceive differences in the shape of the flowers is not accounted for by this study, but, when assessed at the subtribe level, bee-UV-green coloration is shown to be a predictor of shape in 78 per cent of Oncidiinae species, and bee-UV-green orchids are generally more similar to Malpighiaceae than are other plants. The available data did not allow estimation of intra-individual or intra-species shape variation, but the large number of species included does provide an indication that there is a degree of shape convergence across the subtribe. Given the floral plasticity observed within Oncidiinae [27,28,31], it is unsurprising that there is considerably more overlap in shape than in colour. It has been suggested that variation in floral shape is of benefit in deceptive pollination systems, because it prevents pollinators from learning to avoid unrewarding flowers [59]. This could explain why variation in floral morphology has been found to be higher in deceptive species compared with those providing a reward [35]. However, alteration of floral shape has also been shown to reduce pollinator visitation in deceptive species [22,60], and there is probably a dynamic balance operating between these two opposing phenomena.

Within the context of pollinator deception by orchids, several studies have shown the importance of morphological traits in pollination success [61]. Studies of floral shape have mostly focused on sexually deceptive orchids ([62] and references therein). Empirical evidence for the importance of floral shape in insect pollinator attraction is available for many diverse angiosperm and insect taxa [3,62,63]. More specifically, floral shape discrimination by bee pollinators, according to both innate and learned shape preferences, has been demonstrated through behavioural experiments [63]. Thus, given the strong and direct impact of pollinator attraction and pollinator fecundity on plant reproductive success, there is clearly potential for such preferences to result in selective pressures influencing floral morphology.

### (c) Floral mimicry

Convergence on a similar floral phenotype to attract pollinators is clearly only of benefit if the same pollinators are actively being attracted. In three sites, the floral colour of bee-UV-green orchids and Malpighiaceae is distinct from the rest of the community, an indication that neither group exploits non-model mimicry of the general community. Attempts to observe a shared pollinator between Oncidiinae orchids and Malpighiaceae were unsuccessful, highlighting the difficulties in identifying pollinators of deceptive orchids in tropical environments. With such low levels of reproductive success, a longer observation time frame than that of the current study would be needed to confirm shared pollination. Despite this, documentation of a shared pollinator has been recorded between bee-UV-green Oncidiinae and Malpighiaceae. Solitary oil-collecting bees in the family Apidae are known to be important pollinators of many plant species in the Neotropics, including *B. crassifolia* and other Malpighiaceae, as well as a number of Oncidiinae orchids, including *T. ascendens* [29,37,40,56,57]. In some sites, additional bee-UV-green species were present, namely *Vochysia* sp. (Vochysiaceae) and *Acemella* sp. (Asteraceae). These species do not appear to exploit Malpighiaceae pollinators as neither of these genera are primarily pollinated by oil-collecting bees, although occasional visits from *Centris* species have been documented [64,65].

The shared colour signal, convergence in floral shape and potentially shared suite of pollinators raise the possibility that Batesian mimicry may operate between bee-UV-green Oncidiinae mimics and Malpighiaceae models, but other criteria would need to be fulfilled to confirm this [19,23]. The convergence observed has to be driven by the sensory and cognitive abilities of pollinators [24,66]. It is hard to disentangle Batesian floral mimicry from other forms of convergent evolution as the shared environmental conditions and pollinators of both model and mimic would exert similar selective pressure on many floral features [22,67,68]. For convergence on floral colour and shape to be considered a Batesian mimicry system, there needs to be evidence of improved reproductive success in the presence of a model species. Our limited data suggest that this is the case. However, given that pollination success fluctuates between populations for a variety of reasons (e.g. pollinator density [16] or the magnet-species effect [7,69]), further studies need to be carried out to investigate whether there is a general pattern of increased fitness in the presence of a rewarding model. Carmona-Díaz & García-Franco [32] reported higher levels of reproductive success in the Mexican species of *Trichocentrum luridum* (published as *Trichocentrum cosymbephorum*) when occurring in sites where *Malpighia glabra* (Malpighiaceae) was abundant. This phenomenon was also observed in the deceptive orchid *Disa nivea* in South Africa and its Scrophulariaceae nectar-producing model, *Zaluzianskya microsiphon* [21]. Given the generally low reproductive success of deceptive tropical orchids [15,18], this can be difficult to ascertain.

We also assessed whether there was lower frequency of Oncidiinae orchid flowers relative to those of Malpighiaceae, as this is often considered to be an additional criterion for Batesian mimicry. Across two sites, fewer than 250 *T. ascendens* flowers were observed, whereas 150 000–878 000 flowers have been counted on single *B. crassifolia* individuals, indicating that Malpighiaceae flowers are found in greater abundance compared with those of Oncidiinae orchids.

To confirm whether this interaction constitutes strict Batesian mimicry, additional observations of pollination of both plants by the same pollinators would be necessary, as well as extensive testing of increased reproductive success in the presence of the model. Behavioural choice experiments, e.g. [70,71], demonstrating that the pollinator actively confuses the putative model and mimics, both initially and after non-rewarding visits, would further support this hypothesis.

### (d) Non-model deception

Orchids that do not possess a bee-UV-green labellum (e.g. *Oncidium cariniferum*, *Oncidium dichromaticum* (rose/white forms) and *Cuitlauzina convallarioides*) are not distinct from other co-flowering species in any sites. They may exploit mistakes by generalist pollinators, those straying from nearby rewarding species, their perceptual biases or the naivety of early/late season pollinators with innate preferences [72]. As opposed to mimicking a particular model species, this could occur via non-model deception [73] or the magnet-species effect [7,69]. The majority of Oncidiinae exhibiting non-model deception were bee-blue-green in colour (figure 2), which was shown to be the most common flower colour in a study of 593 plant species [55]. Species of this colour made up 33 per cent of the total number of species co-flowering in the 23 study sites. These figures are consistent with non-model deception, as the most beneficial flower colours to imitate would be the most common colours that foraging hymenoptera encounter. Surveys of deceitful orchids in the Mediterranean and Caribbean have concluded that this form of deception is more common than specifically mimicking a single model species [19]. Alternatively, some bee-blue-green Oncidiinae may be mimics of other malpig colour forms, as is thought to be the case between *T. luridum* and *M. glabra* [32].

## 5. Conclusions

We suggest that the bee-UV-green deception characterized here is more complex than a single pair-wise mimetic system. Oncidiinae, many of which possess the mimetic phenotype, are exclusively Neotropical [26–28,33]. The majority of Malpighiaceae occur in the Neotropics [74] and floral conservatism is extremely high within the family—more than 1000 Neotropical species share a floral morphology that attracts oil-collecting bees [40,74]. *Byrsonima crassifolia*, in particular, is dominant throughout the dry forests, savannahs and pastures of Costa Rica [36,75]. The principal pollinators of both groups (oil-gathering bees) are also Neotropical [33]. This suggests that *T. ascendens*, *O. nebulosum* and other mimicking Oncidiinae may not target a single model species; rather, they may attract pollinators from a suite of highly similar Malpighiaceae.

A minority of Oncidiinae produce oil-rewards and studies comparing the oil composition of certain Malpighiaceae and oil-secreting Oncidiinae revealed that there is a high degree of biochemical convergence between the oils [30]. This presents the possibility that non-rewarding species may attract pollinators from both Malpighiaceae and other orchids, thus adding to the complexity of the system. Selection on floral traits may still be driven by the need to attract specific pollinators away from specific rewarding species, but the actual pollinators attracted and the species they are attracted away from may vary in time and space, in a manner akin to the geographical mosaic of coevolution [76]. This could be considered as a multifarious form of Batesian mimicry and may help prevent extinction of these orchids if the mimic's model or pollinators become locally extinct.

These results advance our understanding of reproductive systems underpinning the success of one of the most species-diverse Neotropical groups of plants, namely, the orchids.
